# Validation of albuminuria as a surrogate endpoint in chronic kidney disease: clinical context and implications

**DOI:** 10.1093/ckj/sfag054

**Published:** 2026-02-24

**Authors:** Anna Matyjek, Beatriz Fernández-Fernández

**Affiliations:** Department of Internal Diseases, Nephrology and Dialysis, Military Institute of Medicine – National Research Institute, Warsaw, Poland; Clinical Trials Support Unit, Medical University of Lodz, Lodz, Poland; Department of Nephrology and Hypertension, IIS-Fundacion Jimenez Diaz UAM, Madrid, Spain; Departamento de Medicina, Facultad de Medicina, Universidad Autónoma de Madrid, Madrid, Spain; RICORS2040, Madrid, Spain

Chronic kidney disease (CKD) affects ≈850 million people worldwide and is a rapidly growing cause of morbidity and mortality, with projections placing it among the five leading causes of death globally within the next 15 years [1]. This trajectory highlights the urgent need for effective disease-modifying therapies.

Demonstrating the efficacy of new interventions in CKD traditionally relies on clinical outcomes that are directly meaningful to patients, such as kidney failure or death. However, CKD progression is typically slow and trials powered for these outcomes require long follow-up, large sample sizes and considerable resources. These constraints are particularly limiting in early CKD, where interventions may offer the greatest long-term benefit but where hard endpoints are infrequent. As a result, there is growing interest in surrogate endpoints that can reliably predict clinical benefit while enabling shorter and more feasible clinical trials.

Surrogate endpoints are biomarkers or intermediate outcomes that sit within the causal pathway of disease progression and are intended to capture treatment effects on clinical endpoints. For regulatory acceptance, surrogate endpoints must meet stringent criteria, including biological plausibility, consistent associations with clinical outcomes in observational studies and—critically—trial-level evidence that treatment-induced changes in the surrogate predict effects on clinical endpoints. Such validation typically relies on meta-analytic evidence from randomized controlled trials (RCTs) and includes demonstration of an association between the surrogate and clinical outcomes, quantification of the strength of this association, confirmation of consistency across patient subgroups and interventions and identification of clinically meaningful thresholds for the surrogate endpoint.

In CKD, the slope of the estimated glomerular filtration rate (eGFR) is an established surrogate endpoint [2], accepted by regulatory agencies. However, reliable estimation of the eGFR slope still requires relatively long follow-up. Albuminuria, measured as the urine albumin:creatinine ratio (UACR), has therefore emerged as a complementary candidate surrogate endpoint. Albuminuria reflects glomerular injury and contributes directly to tubulointerstitial inflammation and fibrosis, placing it within the causal pathway of CKD progression. Importantly, changes in UACR can be detected over much shorter time horizons than changes in eGFR, particularly in earlier CKD stages, making it attractive for early-phase RCTs.

In this context, the CKD Clinical Trials Consortium recently published a comprehensive validation of albuminuria as a surrogate endpoint for kidney outcomes [3]. This individual-participant data meta-analysis integrated results from 48 RCTs involving 85 681 participants and 12 therapeutic interventions across a broad spectrum of CKD severities and aetiologies, including CKD in patients with and without diabetes and immunoglobulin A nephropathy (IgAN) (Table 1).

**Table 1:** Overview of study design and findings with clinical interpretation.

Study design/key findings		Clinical explanation
Methods		
Study design	Individual participant data meta-analysis followed by trial-level meta-regression	Surrogate endpoint validation through linkage of treatment effects on UACR with clinical outcomes
Population	CKD with and without diabetes, IgAN	Broad CKD aetiologies represented
RCT treatment interventions	RAASi, SGLT2i, ns-MRA, GLP-1 RA, ETA, immunosuppression regimens	Guideline-directed and novel treatments represented
RCT comparators	Placebo or standard of care	
Surrogate endpoint	Change in UACR at 6 months	Early biomarker evaluated for its ability to predict long-term kidney outcomes
Clinical endpoint	Kidney failure (eGFR <15 ml/min/1.73 m^2^, dialysis or kidney transplantation) or sustained doubling of serum creatinine	Regulatory-accepted hard kidney outcomes in CKD
Results		
Population characteristics	48 RCTs: 85 681 patients, mean eGFR 61.9 ± 46.9 ml/min/1.73 m^2^, median UACR 257 mg/g (IQR 25–970)	Broad population with wide range of kidney function and albuminuria severity represented
Association	30% reduction in UACR to 19% lower hazard of the clinical endpoint	Translation of early albuminuria reduction into renal risk reduction
Strength of association	Median trial-level *R*^2^ = 0.66 (moderate–strong surrogate), Bayesian credible interval 0.06–0.98	Two-thirds of treatment effect on renal outcomes explained by UACR, notable variability (sources detailed below)
Threshold for benefit	≥ 30% UACR reduction to >97–99% probability of renal benefit	Pragmatic target for trials and clinical interpretation
Consistency across CKD subgroups	No major heterogeneity across CKD severity, sex or aetiology; strongest in IgAN	Subgroup consistency, supporting the use of UACR as a surrogate endpoint
Consistency across intervention classes	Proportion of effect explained by UACR: RAASi 82%, SGLT2i 45%, immunosuppression 41%	Variation in effect, restricting applicability of UACR to drugs acting through albuminuria pathways
Limitations	Reduced applicability in non-albuminuric CKD and for therapies not primarily targeting albuminuria; residual renal risk persists; underrepresentation of elderly individuals and women	Context-specific applicability as a surrogate rather than universal replacement of hard endpoints
Implications		
Research	An early ≥30% reduction in UACR may serve as a robust predictor of reduced risk of kidney failure in albuminuric CKD and for interventions targeting albuminuria-mediated mechanisms	Suitability of UACR as a surrogate endpoint in selected settings: albuminuric CKD and albuminuria-mediated interventions
Clinical practice	Early decrease in UACR—a good predictor of reduced risk of kidney failure	Utility for early treatment response assessment and treatment guidance but not as a stand-alone biomarker

ETA: endothelin A receptor antagonist; GLP-1 RA: glucagon-like peptide-1 receptor agonist; ns-MRA: non-steroidal mineralocorticoid receptor antagonist; RAASi: renin–angiotensin–aldosterone system inhibitor; SGLT2i: sodium–glucose cotransporter-2 inhibitor.

Across all trials, active interventions reduced UACR by an average of 25% compared with controls, with consistent effects across CKD stages, aetiologies and sex-specific groups. In parallel, these interventions reduced the risk of the composite clinical endpoint—defined as the composite of kidney failure (eGFR <15 ml/min/1.73 m^2^, initiation of chronic dialysis or kidney transplantation) or doubling of serum creatinine—by ≈24%. Trial-level meta-regression demonstrated a robust association between early, 6-month UACR reduction and clinical benefit: each 30% reduction in UACR was associated with a 19% lower risk of the clinical endpoint, with a probability >99% that this association was true. The surrogate relationship was consistent across CKD aetiologies and appeared particularly strong in IgAN (Table 1).

Importantly, early changes in UACR accounted for, on average, approximately two-thirds of the variability in treatment effects on clinical outcomes, although the precision of this estimate was limited, reflecting substantial variability. For example, the proportion of kidney-protective effects explained by UACR reduction differed by drug class. Therapies acting primarily through albuminuria-mediated pathways, such as renin–angiotensin–aldosterone system blockade or endothelial receptor antagonist, showed that a UACR reduction explained most of the observed benefit (82%). In contrast, for sodium–glucose cotransporter 2 inhibitors or immunosuppressive agents, UACR reduction accounted for only part of the kidney-protective effect (45% and 41%, respectively), indicating contributions from additional mechanisms. These results suggest that UACR is a reasonable surrogate endpoint primarily in patients with albuminuria and for interventions targeting albuminuria-related pathways.

From a clinical perspective, the finding that an absence of UACR reduction is associated with a very low probability of subsequent clinical benefit reinforces UACR as a meaningful treatment target and marker of therapeutic response, as well as a sustained ≥30% reduction in albuminuria within 6 months is a sign of kidney protection, especially in albuminuric CKD. These conditions are relevant for early clinical decisions but do not replace long-term outcome follow-up and eGFR monitoring [3, 4].

For trialists, identifying a 30% early reduction in UACR as a threshold linked to a high probability of clinical benefit provides actionable guidance for the design of future studies, particularly early-phase RCTs.

Overall, this work offers a framework for surrogate endpoint validation in CKD and demonstrates that early albuminuria reduction can accelerate therapeutic development while preserving relevance to patient-centred outcomes in albuminuric CKD and for interventions acting through albuminuria-mediated pathways, with the strongest effects observed in IgAN.

## CONFLICT OF INTEREST STATEMENT

The authors declare no conflict of interest regarding the publication of this paper.
